# Supplementary material for: Antagonistic roles for Ataxin-2 structured and disordered domains in RNP condensation
Source: eLife. 2021 Mar 10;10:e60326. doi: 10.7554/eLife.60326 (PMC7946432; doi:10.7554/eLife.60326)
Supplement: Supplementary file 1. [file elife-60326-supp1.docx]

**Supplementary File 1:** Primers used in the study.

| **Primer Name** | Sequence 5’ to 3’ | Purpose |
| --- | --- | --- |
| Atx2 ORF F | AATACAAGAAGAGAACTCTGAATAATGAACAACAATAGCAAGCGGAAAAC | Whole CDS amplification |
| Atx2 ORF F | TACGGCGGTGGCAGGCCTTGAATGGAC | Whole CDS amplification |
| Lsm F | TGGGATTCTGGCGCTAATGG | Lsm domain deletion |
| Lsm R | AAACGTGTTGTTGTAGACGCC | Lsm domain deletion |
| LsmAD F | TCGACCGAGCAGGATCAACG | LsmAD domain deletion |
| LsmAD R | CGTGTTCTCATTTTTGCGGAAC | LsmAD domain deletion |
| mIDR F | CACGTGGTCCTGCACCAAGT | mIDR domain deletion |
| mIDR R | GTTGCGCATCACCTTACCGC | mIDR domain deletion |
| cIDR F | GGAGGACATGTACCGgcCACCGCGGTGgACTAC | cIDR domain deletion |
| cIDR R | CGGTACATGTCCTCCCGTGG | cIDR domain deletion |
| Lsm+LsmAD F | AGAAGAGAACTCTGAATAGATCTATGTTCATGCACTCGGCCACGGC | Only Lsm+LsmAD domain |
| Lsm+LsmAD R | CACCACCATTGGTGAAGGGGCGGCCGCTGGACGCTCCACGGCCGCG | Only Lsm+LsmAD domain |
| 2XLsm F | GGTAAGGTGATGCGCAACTTCATGCACTCGGCCACGG | Additional Lsm domain in place of mIDR |
| 2XLsm R | TTGGTGCAGGACCACGTGTGGTTCCAATTCCTTCTCGTCCG | Additional Lsm domain in place of mIDR |
| Mock Forward | TAATACGACTCACTATAGGGATGGTGAGCAAGGGCGAGGAG | IVT of mock dsRNA |
| Mock Reverse | TAATACGACTCACTATAGGGCTTGTACAGCTCGTCCATGCCG | IVT of mock dsRNA |
| Atx2 Forward | TAATACGACTCACTATAGGGCCGGTGGTTAAGAAGCATGT | IVT of Atx2 dsRNA |
| Atx2 Reverse | TAATACGACTCACTATAGGGGAATTGTTGCTGGTGTGGTG | IVT of Atx2 dsRNA |
